# Differences in Transcription Patterns between Induced Pluripotent Stem Cells Produced from the Same Germ Layer Are Erased upon Differentiation

**DOI:** 10.1371/journal.pone.0053033

**Published:** 2013-01-09

**Authors:** Iryna Pirozhkova, Ana Barat, Petr Dmitriev, Elena Kim, Thomas Robert, Justine Guégan, Chrystèle Bilhou-Nabera, Florence Busato, Jörg Tost, Gilles Carnac, Dalila Laoudj-Chenivesse, Marc Lipinski, Yegor Vassetzky

**Affiliations:** 1 CNRS, Université Paris-Sud, UMR-8126, Institut de cancérologie Gustave Roussy, Villejuif, France; 2 Laboratory of Epigenetics, Centre national de génotypage CEA-Institut de génomique, Evry, France; 3 Laboratory for Functional Genomics, Fondation Jean Dausset – CEPH, Paris, France; 4 Integrated Biology Platform, Institut de cancérologie Gustave Roussy, Villejuif, France; 5 INSERM U1046, Université Montpellier 1, Université Montpellier 2, Montpellier, France; CNRS, France

## Abstract

Little is known about differences between induced pluripotent stem cells produced from tissues originating from the same germ layer. We have generated human myoblast-derived iPS cells by retroviral transduction of human primary myoblasts with the *OCT3/4*, *SOX2*, *KLF4* and *MYC* coding sequences and compared them to iPS produced from human primary fibroblasts. When cultivated *in vitro*, these iPS cells proved similar to human embryonic stem cells in terms of morphology, expression of embryonic stemness markers and gene promoter methylation patterns. Embryonic bodies were derived that expressed endodermal, mesodermal as well as ectodermal markers. A comparative analysis of transcription patterns revealed significant differences in the gene expression pattern between myoblast- and fibroblast-derived iPS cells. However, these differences were reduced in the mesenchymal stem cells derived from the two iPS cell types were compared.

## Introduction

The induced pluripotent stem (iPS) cells approach has opened unprecedented possibilities for the generation of patient-specific pluripotent stem cells useful for studies on the origin of various pathologies as well as for possible corrections of genetic defects and cell transplantation therapies [1] [2], [3]. iPS cells now represent a potentially inexhaustible cell resource with a differentiation potential similar to that of embryonic stem (ES) cells. Somatic cells can be reprogrammed to a pluripotent state by transduction of either *OCT4, SOX2*, *NANOG*, and *LIN28* [4] or *OCT4*, *SOX2*, *KLF4* and *MYCs* [5], [6] coding sequences, or alternatively by RNA or protein transduction approaches [7]. iPS cells resemble ES cells in terms of epigenetic and gene expression patterns, morphology, and ability to differentiate into endodermal, mesodermal and ectodermal derivatives [4], [5], [6], [8].

Up till now, human iPS cells have been generated mostly from parental fibroblasts of diverse origin [6], [9], [10], keratinocytes [8] and mesenchymal stromal cells [11]. In humans, iPS cells have recently been obtained from both differentiated human muscle and from skeletal muscle stem cells [12], [13]. We have independently produced induced pluripotent stem cells from human primary myoblasts which specifically expressed CD56 at their cell surface and were isolated from muscle biopsies prior to being maintained in cell culture. In conditions of serum starvation, these primary myoblasts differentiated into myotubes under the control of a core transcriptional network determined by myogenic regulatory factors including *MYF5*, MYOD, myogenin and MRF4 [14], [15].

Earlier studies had indicated that iPS cells retained the transcription memory of the germ layer origin of their parental somatic cells [16], [17] [18]. It was not clear, however, whether iPS cells generated from parental cells sharing the same germ layer origin would display a common expression pattern. In the present paper, we compare gene expression in differentiating iPS cells produced from either myoblasts or fibroblasts.

## Materials and Methods

All cell lines used in this study are presented in **Table S1**.

### Myoblast isolation and culture

Human primary myoblasts were isolated from skeletal muscles (quadriceps) as described in [14], purified with immuno-magnetic sorting system (Miltenyi Biotec, USA) using an anti-CD56/NCAM antibody (BD Biosciences) according to the manufacturer's specifications. CD56-positive myoblasts were seeded in collagen-coated Petri dishes (P1) and cultured in DMEM, supplemented with 10% foetal calf serum, 1% Ultroser G (Biosepra), at 37°C with 5% CO_2_. Myoblast purity was determined by staining for desmin (Sigma). The cells were used at passage 1 to passage 3 for the generation of iPS cells.

### Fibroblast isolation and culture

Human normal adult fibroblasts (Coriell cell repository) were maintained in DMEM high glucose supplemented with 10% foetal calf serum, 1 mM sodium pyruvate (Invitrogen) and 2 mM glutamax (Invitrogen).

### iPS cell culture

iPS cell lines from primary human fibroblasts were previously obtained and characterized at the Institute for Stem cell Therapy and Exploration of Monogenic Diseases, (I-Stem, Evry France) [19].

iPS cell lines from primary human myoblasts were generated in this work in collaboration with I-Stem (Evry France) by infection with retroviral cassettes harbouring the cDNA encoding *OCT4*, *SOX2*, *c-MYC* and *KLF4*, and *GFP* under transcriptional control of its promoters (Addgene,Cambridge, MA) (Addgene plasmids 17220, 17225, 17226, 17227). These plasmids were individually transfected using FuGene (Roche) into PLAT-A (for amphotropic viral production) packaging cells. PLAT cells medium was replaced 24 hours post-transfection. Viral supernatants were collected 48 hours post-transfection, filtered through a 0.45 µm filter, then mixed at a 1∶1∶1∶1 ratio.

iPS cells were cultured either on mouse embryonic fibroblasts (MEF) prepared from E14 mouse embryos or on human foreskin fibroblasts (BJ1) feeder cells which were mytomycin-C growth-arrested. BJ1 cells express GFP and FGF2 protein were perepared at the iSTEM platform. hES culture medium was KO/DMEM (Invitrogen) supplemented with 20% knockout serum replacement (KSR) (Invitrogen), 0.1 mM nonessential amino acids (Invitrogen), 2 mM glutamax (Invitrogen), 50 µM β-mercaptoethanol (Invitrogen), 100 UI/ml penicillin/streptomycin (Invitrogen). hES cell medium for MEF feeder was supplemented by 10 ng/ml fibroblast growth factor FGF2 (Invitrogen). The iPS cells were passaged every 7 days.

### Retroviral Transduction

Cryovial of Platinum-A (PlatA) cells (Cell Biolabs) were used for transient virus packaging. 3×10^6^ PlatA cells were plated per 60 mm gelatine-coated dish (80% confluent) in PlatA medium of DMEM+Glutamax II (Invitrogen) containing 10% foetal calf serum, 1 mM sodium pyruvate (Invitrogen) and 50 mM β-mercaptoethanol. After 24 h incubation pMYG retroviral vectors containing hOCT4, hSOX2, hKLF4, hcMYC and GFP were transfected into PlatA cells with FuGENE HD transfection reagent (Roche). After 48 h viral supernatants were collected, filtered in the tubes with polybrene/HEPES mixture. Adult somatic cells were infected with a mixture of viral supernatant containing each reprogramming factors in equal quantity. The transduction efficiency was checked by expression of GFP FACS analysis (MACSQuant of Miltenyi).

### Generation of iPS Cells from Myoblasts

Four days before the transduction, 2.5×10^4^ cells or 50×10^4^ cells were seeded onto 25 mm plates. One day before retroviral infection, the myoblast cells were seeded at 10^5^ cells per well in 6-well plates. The viral supernatant was added only one as it was sufficient. One day after transduction the cells were seeded in 6-well collagen-coated plates at different dilutions: 5×, 10×, 30×, 40× and 80×, in the myoblast medium. After 24 h the myoblast medium was replaced with hES cell medium supplemented with 10 ng/ml FGF2 and 0.5 mM valproic acid (VPA) (Sigma-Aldrich) for 10 days. The medium was replaced every day and VPA has been omitted from culture medium from day 11. Around 3–5 weeks after viral reprogramming, iPS colonies were picked every day on the basis of ES cell-like morphology. The iPS colonies were transferred onto BJ1-FGF2 feeder plates and maintained in hES cell medium. ROCK inhibitor (Calbiochem) was added at 10 µM during the first three days to enhance survival of dissociated iPS cells.

### MSC Differentiation

The iPS cells were directly differentiated into MSC cells by serum induction. The iPS cells were incubated in MSC medium containing KO/DMEM (Invitrogen) supplemented with 20% FCS, 0.1 mM nonessential amino acids (NEAA) (Invitrogen), 2 mM glutamax, 50 µM β-mercaptoethanol, 100 UI/ml penicillin/streptomycin (Invitrogen). The medium was changed every 2–3 days. FGF2 (10 ng/ml) and Vitamin C (1 mM; Sigma) were added up to the first passage. After passages P4–P5, cells were seeded at 4000cells/cm^2^.

### Embryoid Body Formation

Human iPS cells were treated with collagenase (Invitrogen), harvested and transferred to low attachment culture 6-well plates (NalgeNunc) in hES cell medium without FGF2. These cell aggregates were allowed to grow for several days or weeks and samples were harvested at various time points for differentiation markers analysis.

### RNA Isolation and Reverse Transcription

Total RNA was extracted using the Qiagen RNA-easy Kit from iPS cells at passages 22–25, from MSC at passages 6 and from primary myoblasts at passage 6. cDNA was synthesized from 500 ng of total RNA using RevertAid H Minus First Strand cDNA Synthesis Kit (Fermentas) for RT-PCR analysis. PCR primer sequences are shown in **Table S2**. Total RNA was isolated for transcriptome analysis using Trizol (Invitrogen), according to the manufacturer's instructions.

### Bisulfite Pyrosequencing Analysis

Genomic DNA was isolated using the Wizard SV Genomic DNA purification system (Promega). Quantitative DNA methylation analysis was performed by pyrosequencing of bisulfite-treated DNA [20]. 500 ng of DNA was bisulfite converted using the EpiTect 96 Bisulfite kit (Qiagen, Hilden, Germany) according to the manufacturer's instructions. Two regions in *OCT4* and one in *NANOG* were amplified using 30 ng of bisulfite-treated human genomic DNA and 5 to 7.5 pmol of forward and reverse primers, one of them being biotinylated. Oligonucleotide sequenes for PCR amplification and pyrosequencing are given in Table S3. Reaction conditions were 1× HotStar Taq buffer supplemented with 1.6 mM MgCl_2_, 100 µM dNTPs and 2.0 U HotStar Taq polymerase (Qiagen) in a 25 µl volume. The PCR program consisted of a denaturing step of 15 min at 95°C followed by 50 cycles each of 30 s at 95°C, 30 s at the respective annealing temperature and 20 s at 72°C, with a final extension step of 5 min at 72°C. 10 µl of PCR product were rendered single-stranded as previously described [20] and 4 pmol of the respective sequencing primers were used for analysis. Quantitative DNA methylation analysis was carried out on a PSQ 96MD system with the PyroGold SQA Reagent Kit (Pyrosequencing). Results were analyzed using the Q-CpG software (V.1.0.9, Pyrosequencing AB).

### Alkaline Phosphatase Staining and Immunocytochemistry

iPS cells were washed with PBS, fixed in 4% paraformaldehyde in PBS for 15 minutes, then rinsed with PBS. To allow nuclear permeation, the cells were treated with 50 mM NH_4_Cl (Sigma) for 10 minutes, rinsed with PBS and treated with 0.2% Triton X-100 (Sigma) in PBS for 4 minutes. After PBS washes, cells were blocked in 0.5% bovine serum albumin (Sigma) in PBS for 30 minutes. Cells were stained with the following primary antibodies: hOCT3/4 (1∶200; goat; Santa Cruz Biotechnology); SSEA-4 (1∶100; goat; BD Biosciences), TRA-1-60 (1∶100; mouse; Millipore), TRA-1-81 (1∶100; mouse; Millipore). Appropriate Alexa Fluor 488-conjugated secondary antibodies (Invitrogen) were used at a 1∶1,000 dilution. DAPI was added at 0.1 ng/ml. Alkaline phosphatase staining was performed according to manufacturer's instructions (Sigma Aldrich).

### Flow cytometry analysis

The following PE-conjugated human monoclonal antibodies were used for the flow cytometry analysis at 1∶5 dilutions: the iso-IgG1 (BD Biosciences), anti-CD29 (BD Biosciences), anti-CD44 (BD Biosciences), anti-CD73 (BD Biosciences), anti-CD105 (Abcys), and anti-CD166 (BD Biosciences). The samples were analyzed on MACSQuant (Miltenyi).

### Karyotype Analysis

Karyotyping analysis was performed on every iPS cell line at passage 22. Actively growing iPS cell colonies were treated with colchicine at 20 mg/ml (Eurobio) for 90 minutes at 37°C. After washing, cells were incubated in trypsin–EDTA 0.05% (Eurobio) for 2–3 minutes and harvested. Cells were incubated in 75 mM KCl (Sigma) for 10–14 minutes at 37°C, followed by fixation with 3∶1 methyl alcohol/glacial acetic acid. Fixed cells were dropped on wet slides and dried at 37°C for 24 hours. Chromosomal G-band analysis and multicolour FISH were performed at Kremlin Bicetre Hospital.

### Microarray Expression Analysis

The quantity and purity of the extracted RNA was evaluated using a NanoDrop spectrophotometer. Its integrity measured using an Agilent Bioanalyzer. For microarray hybridizations, 500 ng of total RNA from each RNA sample was amplified and labelled with fluorescent dye (Cy3) using the Low RNA Input Linear Amplification Labelling kit (Agilent Technologies, Palo Alto, CA, USA) following the manufacturer's protocol. Cy3-labeled cRNA were hybridized to the Agilent Human 44K Whole Genome Oligo Microarray (Agilent Technologies), prior to washing and scanning. Data were extracted from scanned images using Feature Extraction software (v 10.5.1.1 Agilent) with default settings.

### Gene Expression Analysis

Gene expression analysis was carried out using R and Bioconductor [Gentleman 2004]. After removal of probes with an intensity lower than background in more than two samples, the dataset was reduced from ∼41,000 to ∼38,600 probes. Normalization was performed using vsn library [21]: for each array, a scaling factor is found that makes the as similar as possible data across arrays, using least squares regression. The generalised log-transformation is applied to the spot intensities. Differential expression analysis of normalized data was performed using limma package [22], allowing simultaneous comparisons between more than two mRNA targets by fitting a linear model to the expression data for each probe. The genes differentially expressed between fibroblasts and myoblasts, iPS derived from myoblasts (miPS) and MSC derived from miPS respectively (resulting in three contrasts) have been identified using an Empirical Bayesian approach, robust for small numbers of arrays and equivalent to shrinkage of the estimated sample variances towards a pooled estimate, resulting in a stable inference when the number of arrays is small [Smyth 2004]. For each target probe, a moderated t-statistic variable was computed, accompanied by an adjusted P-value obtained with the Benjamini-Hochberg procedure for multiple test adjustment. Sets of genes with adjusted P-value<0.05 and log(Odds(Probe differentially expressed))>0 were selected as differentially expressed.

We have included two fiPS samples grown on MEF feeder for analysis of gene expression of a list of muscle-specific genes in fiPS and miPS (Tables 1 and 2). These two samples had a slightly different expression pattern as compared to fiPS grown on human BJ1 feeder. If the absolute log(fold-change) between the expression values of MEF_fiPS and BJ_fiPS stemming from the same cell line was smaller than 1.5, an additional comparison is made between the expression of the three fiPS and four miPS samples, a classical t-test was performed and the corresponding p-value was reported (in Table 2).

**Table 1:** List of genes upregulated in both miPS cells and myoblasts *vs* fibroblasts and sharing muscle descriptors of high score.

Descriptor	GO ID	Genes sharing this descriptor
**Muscle System Process**		**26**
- muscle system process	**GO:0003012**	ACTA1, ACTN2, ATP1A2, CHRNB1, DAG1, DTNA, DYSF, EDNRB, GAA, KCNH2, KCNJ12, KCNQ1, MYH2, MYH6, MYL4, PGAM2, RYR1, SCN5A, SLC6A8, SMPX, SNTB1, TNNC1, TNNC2, TNNT1, TNNT2, TRIM63
**Muscle Contraction**		**27**
- muscle contraction	**GO:0006936**	ACTA1, ACTC1, ACTN2, ATP1A2, CHRNB1, DAG1, DTNA, DYSF, EDNRB, GAA, KCNH2, KCNJ12, KCNQ1, MYH2, MYH6, MYL4, PGAM2, RYR1, SCN5A, SLC6A8, SMPX, SNTB1, TNNC1, TNNC2, TNNT1, TNNT2, TRIM63
- striated muscle contraction	**GO:0006941**	
- skeletal muscle contraction	**GO:0003009**	
- cardiac muscle contraction	**GO:0060048**	
- regulation of muscle contraction	**GO:0006937**	
**Muscle filament sliding**		**11**
- muscle filament sliding	**GO:0030049**	ACTA1, ACTC1, ACTN2, MYH2, MYH6, MYL4, TMOD1, TNNC1, TNNC2, TNNT1, TNNT2
- regulation of muscle filament sliding speed	**GO:0032972**	
- actin-myosin filament sliding	**GO:0033275**	
- actin-myosin structure organisation	**GO0031032**	
**Muscle development**		**16**
- muscle development	**GO:000751**	ACTA1, BMP4, C6orf32, CHKB, CHRB1, FXR1, GAA, JPH1, MLLT7, MYLPF, MYOZ1, NEURL2, OBSL1, PROX1, TEAD4, TMOD1
- striated muscle cell development	**GO:001470**	
- muscle cell development	**GO:0055001**	
- striated muscle development	**GO:0055002**	
- visceral muscle development	**GO:0007522**	
- cardiac muscle fiber development	**GO:0055013**	
- skeletal muscle fibre development	**GO:0048741**	
- ventricular cardiac myofibril development	**GO:005505**	
**Morphogenesis, Differentiation, Assembly and Migration**		**16**
- striated muscle cell differentiation	**GO:0051146**	ACTA1, ACTC1, BMP4, GAA, MLLT7, MYH6, MYOZ1, PDGFA, PDGFB, PDLIM3, NEURL2, OBSL1, PROX1, TMOD1, TNNC1, TNNT2
- ventricular cardiac muscle morphogenesis	**GO:0055010**	
- cardiac muscle tissue morphogenesis	**GO:0055008**	
- muscle tissue morphogenesis	**GO:0060415**	
- muscle cell differentiation	**GO:0042692**	
- atrial cardiac muscle morphogenesis	**GO:0055009**	
- regulation of muscle cell differentiation	**GO:0051147**	
- skeletal muscle thin filament assembly	**GO:0030240**	
- cardiac muscle thin filament assembly	**GO:0071691**	
- myofibril assembly	**GO:0030239**	
- skeletal myofibril assembly	**GO:0014866**	
- structural constituent of muscle	**GO:008270**	
- smooth muscle cell migration	**GO:0014909**	

**Genes, attributed to muscular phenotype by GeneDecks and SetDistiller:** ACTC1, ADAM17, ALPL, APP, AQP5, AR, ARRB1, ATP2A3, ATRNL1, BIN1, BMP4, BSN, CA3, CDKN1C, CEBPA, CHD2, CHKB, COL14A1, CTNNB1, CXCR7, CYP19A1, DAAM1, DHCR7, DLL1, DOCK3, EGLN3, EPC1, ERBB3, EYA1, FABP3, FXR1, H19, HEY1, HIF3A, HSD11B2, IGF2, JPH1, KCNC1, KCNC3, KCNJ2, KCNJ6, KIF1B, KLF15, MYCN, MYLPF, MYOZ1, NACA, NEDD4L, NEURL2, NRCAM, OBSCN, PDE4D, PDPN, PECAM1, PEX7, POR, PPARGC1A, RB1CC1, RTN2, SLC6A6, SLC7A7, SLN, SMAD1, SMYD1, SYNE2, THRB, TLR2, TMEM27, TMEM38A, TP53, TRIM54.

**Genes, found to have high expression in muscle by GeneDecks and SetDistiller:** ABHD4, ADSSL1, AGL, ALPK3, ASB2, ATP1A2, BIN1, BSG, BTG2, C1orf187, C21orf33, CA3, CACNG6, CCDC3, CFD, COX6C, DDIT4, DNAJB5, DUSP13, ECH1, EIF4A2, ENO3, FKBP5, FNDC5, FXYD6, GADD45G, GATS, H19, HES6, HSD11B1L, IMPA2, ITGB1BP3, KIAA0319L, MFSD3, OBSCN, PABPC1, PAIP2, PDK4, PGAM2, PHYHD1, PPP1R3B, PYGM, RBM17, RBM38, ROGDI, RPL22, RTN2, RYR1, SARS2, SELENBP1, SEPW1, SESN1, SLN, SMPX, SORBS1, TMEM134, TMEM38A, TRIM54, TSPAN7, UCP2, USP54.

**Table 2:** List of genes upregulated in both mMSC and myoblasts *vs* fibroblasts and sharing muscle descriptors of high score.

Descriptor	GO ID	Gene	Myo/fibro fold ch.	miPS/fibro fold ch.	mMSC/fibro fold ch.	miPS/fiPS fold ch.
**Morphogenesis**						
- cardiac muscle tissue morphogenesis	**GO:0055008**	**ANKRD1**	**40 adj.p<0.05**	1.27 adj.p = 0.5, raw.p = 0.41	**18 adj.p<0.05**	**6.45 adj.p<0.05**
**Muscle proliferation and Migration**						
- striated muscle cell proliferation	**GO:0014855**	**TGFB2**	**10.5 adj.p<0.05**	2 adj.p<0.11 **raw.p = 0.07**	**7.4 adj.p<0.05**	3.2 adj.p = 0.2 **raw.p = 0.045**
- cardiac muscle cell proliferation	**GO:0060038**					
-regulation of smooth muscle cell migration	**GO:0014910**	**PDGFA**	**44 adj.p<0.05**	**15.2 adj.p<0.05**	**31.6 adj.p<0.05**	1.6 adj.p<0.45 raw.p = 0.2
- smooth muscle cell migration	**GO:0014909**					
- smooth cell migration	**GO:0014812**					
**Structure**						
- structural constituent of muscle	**GO:000837**	**PDLIM3**	**227 adj.p<0.05**	**8 adj.p<0.05**	**17 adj.p<0.05**	2.3 adj.p<0.59 p = 0.3

**Genes, attributed to muscular phenotype by GeneDecks and SetDistiller tools:** CA3, CHD2, IGF2, ITGAV, ITPR1, LIMS1, MYOZ2, NEDD4L, PDE4D, TLR4.

**Genes, found to have high expression in muscle by GeneDecks and SetDistiller tools:** APBB2, ATF6, CASD1, CCND2, FGFR1OP2, GLIS3, HOXC11, MSI2, MTHFD2L, TDRKH.

When adj.p-value is larger then our significance threshold of 0.05, the unadjusted raw.p-value is also given. Statistically significant fold changes are in bold.

### Functional Analysis

Functional analysis for the differentially expressed genes in the various contrasts was carried out using annotate and GOstats packages from Bioconductor. The hypergeometric P-values for overrepresentation of genes for all GO terms in the induced GO graph were computed. This is based on the idea of comparing the frequency of genes annotated with a particular GO term among the differentially expressed genes with the overall frequency of this particular GO term among all genes represented on the array. Biological Process (BP) GO terms with a P-value<0.1 have been selected. GO terms related to muscular function were selected from the significant BP GO terms.

The gene ontology (GO) terms as well as other system descriptors were also analysed by *Set distiller* feature of *GeneDecks Version 3* analysis tool (http://www.genecards.org), ranked by significance of enrichment, which was denoted by P-value>0.05 and corrected for multiple testing by Bonferroni correction.

## Results

### Generation of iPS cells from purified primary myoblasts

**Figure 1:**
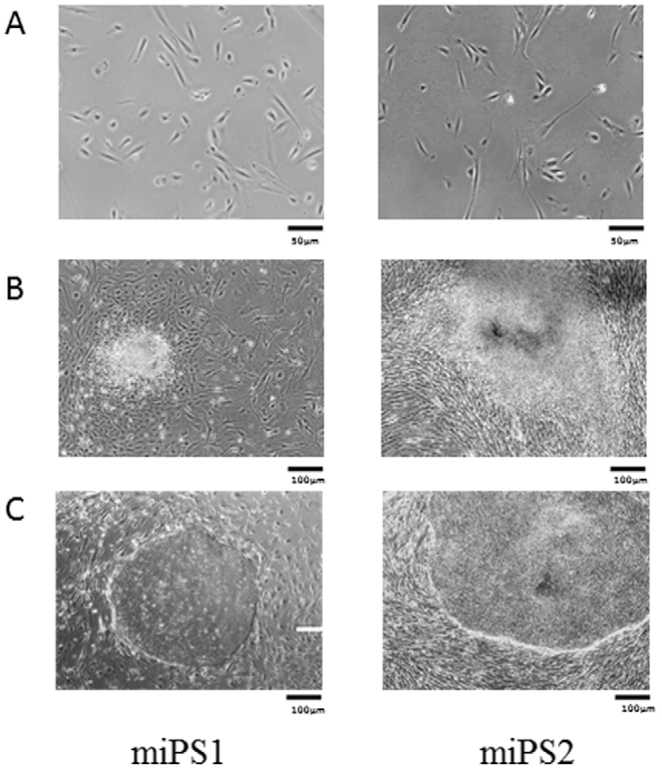
Generation of two independent iPS cell-lines derived from purified CD56+ primary myoblasts. **A**, Purified myoblast cell cultures derived from two healthy individuals; **B**, Emergence of « ES-like » clones observed at day 21 after tranduction; **C**, miPScellseach derived from distinct emerged clones and cultivated for 14 days on a BJ1 cell feeder.

Primary CD56^+^ myoblasts purified from a quadriceps biopsy were infected using retroviral vectors containing human *OCT4*, *SOX2*, *C-MYC* and *KLF4* coding sequences and *GFP* (see Materials and methods). One day after transduction verified by GFP expression, virally-infected cells were seeded into 6-well collagen-coated plates at various dilutions and grown in human ES cell culture medium. The first colonies with a human ES-like morphology were harvested three weeks after reprogramming (**Figure 1**). These colonies were then grown on BJ1 fibroblasts used as an FGF2-producing feeder. In such conditions, iPS cells are maintained in a pluripotent state. They are referred to as myoblast-derived induced pluripotent stem (miPS) cells.

### Characterization of miPS cells

**Figure 2:**
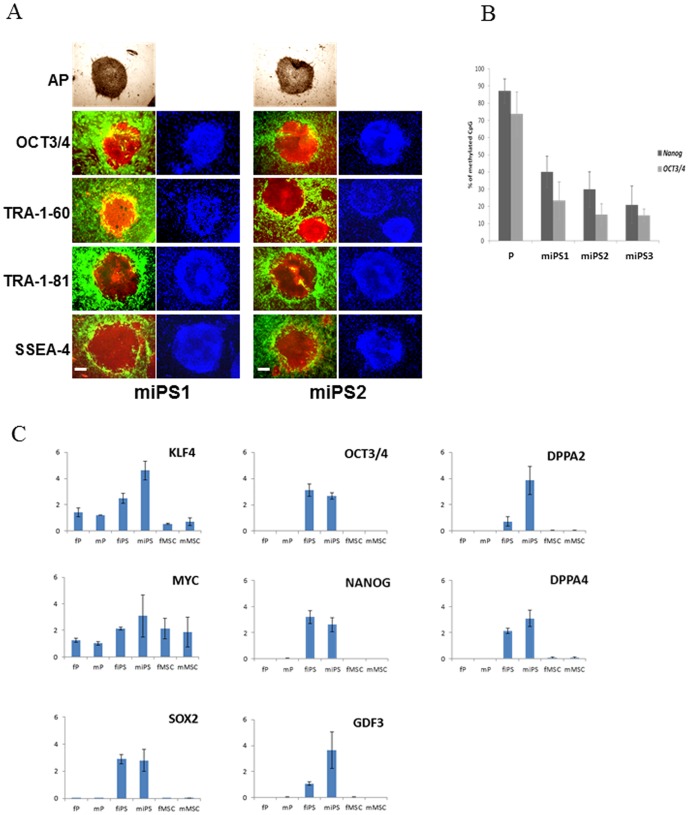
Characterization of human miPS cells. **A**, Immunostaining of established miPS cell clones for pluripotency markers. AP, alkaline phosphatase. Red staining from PE-conjugated human monoclonal antibodies, green from GFP-expressing BJ1 feeder, and blue for DAPI. *Scale bars* = 100 µm. **B**, DNA methylation status of CpG dinucleotides in the *OCT3/4* and *NANOG* gene promoter regions in parental (P) and miPS cells. **C**, Expression levels of pluripotency marker and reprogramming genes in parental (P) cells, iPS cells and iPS-derived MSC of fibroblast (f) and myoblast (m) lineages. Data were extracted from the 44K+ Agilent transcriptome array and scaled. Y axis, expression levels normalized against β-actin gene expression. The data are representative of 3–4 independent experiments. Expression levels of pluripotency marker and reprogramming genes in parental cells (lanes 1–5), iPS cells (lanes 6–11) and iPS-derived MSC (lanes 12–17). Data were extracted from the 44K+ Agilent transcriptome array and scaled. Y axis, expression levels normalized against *β-actin* gene expression. The experiments were carried out in duplicate.

To characterize the clones obtained, ES cell markers were first searched on 10 to 20 colonies immunocytochemically stained. As exemplified for two miPS cell-lines, (**Figure 2A**) all colonies proved positive for the ES cell-specific surface antigens *SSEA-4*, *TRA-1–60* and *TRA-1–81* as well as for the ES cell-specific transcription factor *OCT3/4*, consistent with an iPS cell phenotype. We then investigated the DNA methylation status in the promoter regions of the *OCT3/4* and *NANOG* genes. In the three miPS cell-lines tested, both promoters were found to be less methylated than in parental somatic cells (**Figure 2B**), indicating that both genes had been activated. The miPS1 and miPS2 cell-lines were further analyzed for their karyotypes at passage 22 when both were found to be diploid and normal (**Figure S1**).

### Expression of transgenes and endogenous stemness genes

Next, we examined transcription levels for various stemness marker genes. miPS cells were found to express undifferentiated ES cell marker genes including the reprogramming *KLF4*, *OCT3/4*, *SOX2*, *MYC* and *NANOG* genes, the developmental pluripotency-associated proteins *DPPA2* and *DPPA4* as well as *GDF3* (**Figure 2C** **, lanes 6–11**). This expression was endogenous, all transduced genes being silent with the single exception of the transduced *MYC* gene which participated in total *MYC* expression until the MSC stage when it was found to be totally extinct (**Figure S2**). Untransduced parental cells only expressed *MYC* and *KLF4* (**Figure 2C** **, lanes 1–5**) which were also expressed by the six MSC tested (**Figure 2C** **, lanes 12–17**).

### Spontaneous differentiation of miPS cells

**Figure 3:**
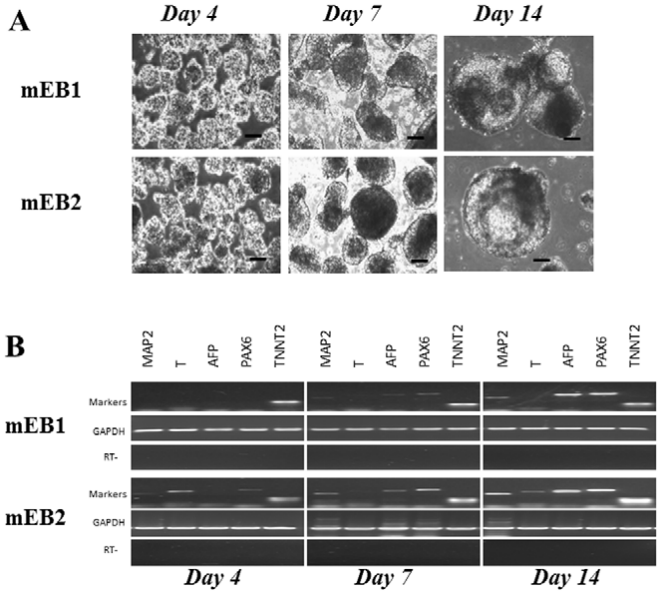
Spontaneous *in vitro* differentiation of miPS cells into embryoid bodies (mEB1, mEB2). **A**, Phase contrast microscopy observation of EBs at days 4, 7, 14. Scale bars = 100 µm; **B**, RT-PCR analysis (30 cycles) of expression of germ layer marker genes. *GAPDH* and RT-, housekeeping gene and negative controls.

In order to examine the potential of miPS cells to differentiate into each of the three germ layers, we used a floating culture approach whereby miPS cells were found to generate spherical embryoid bodies (EB) of spontaneously differentiating cells. As displayed in **Figure 3A**, aggregates were observed as early as day 4, followed by fusion of two or more EBs which formed larger aggregates of differentiated cells as shown at days 7 and 14 in **Figure 3A**. The self-aggregation process was associated with the appearance of different cell types. Markers associated with the three germ layers were expressed progressively at days 4, 7 and 14 in the two EB cell-lines mEB1 and mEB2 (**Figure 3B**). The mesodermal marker gene *TNNT2* was strongly expressed in both lines as early as day 4 while the endodermal marker gene *AFP* and the ectodermal marker genes *MAP2* and *PAX6*, detected at day 7, were more strongly expressed at day 14 (**Figure 3B**).

**Figure 4:**
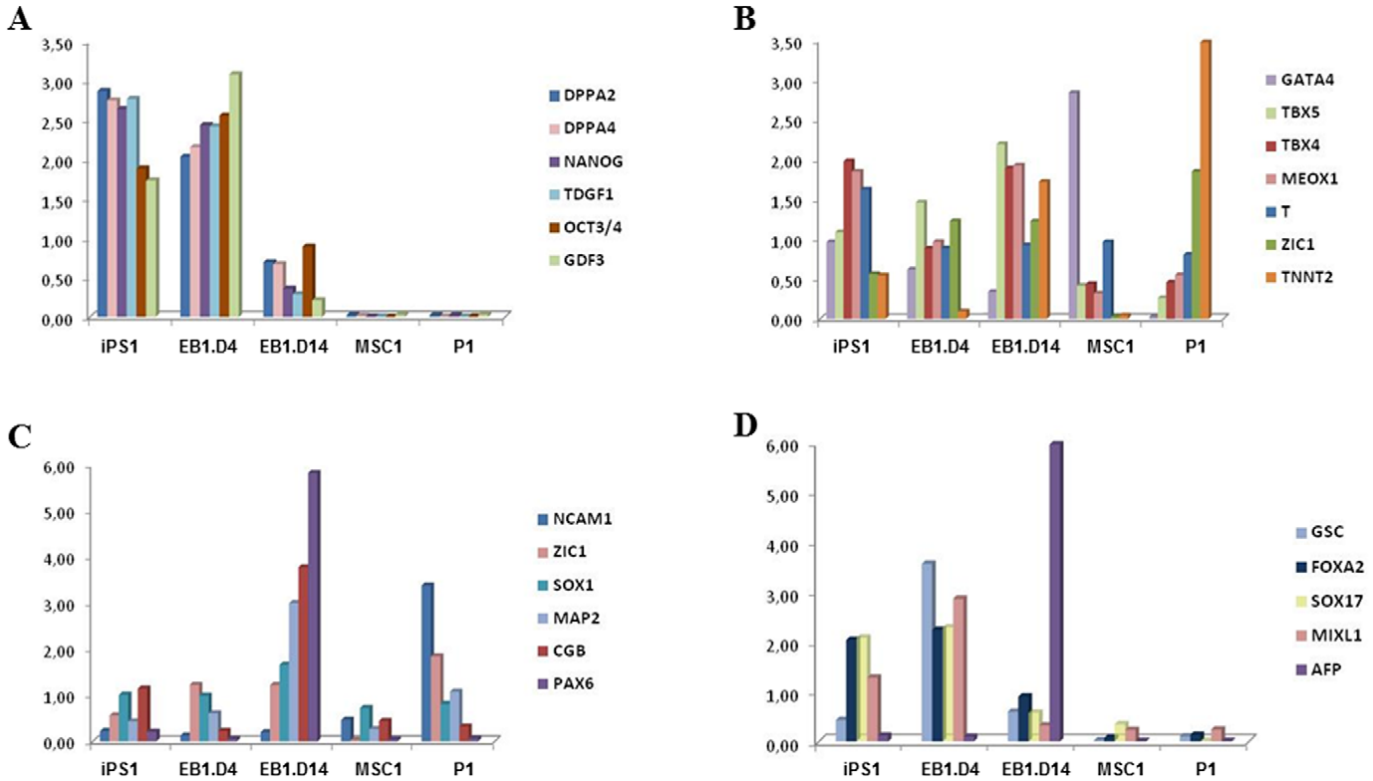
Expression analysis of germ layer-specific markers. Expression patterns of (A) pluripotency, (B) mesoderm, (C) ectoderm and (D) endoderm markers, in parental (P1), myoblast-derived iPS (miPS1), miPS-derived embryonic body (EB1.D4 and EB1.D14) cells and MSC. Data are presented for individual samples. Y axis, expression levels normalized against β-actin expression.

Further analysis was performed on the mEB1 cell-line. Expression levels of pluripotency, mesodermal, ectodermal and endodermal marker genes are displayed in **Figure 4**, panels A, B, C and D, respectively. For comparison purposes, the same analysis was performed on EB1 (EB1.D4 and EB1.D14) as well as on the corresponding parental (P1) and miPS (IPS1) cells and on derived MSC (MSC1). At day 4, EB1 cells were found to express the six stem markers tested as strongly as iPS cells. This expression decreased at day 14 (EB1.D14). Parental cells or MSC were negative (**Figure 4A**). All markers associated with the three germ layers were expressed at days 4 and/or 14 in the EB cell-line (**Figure 4B–D**). In general, mesodermal markers were expressed from iPS to EB and MSC stages of differentiation (**Figure 4B**) to a higher degree than the ectodermal and endodermal markers (**Figure 4C–D**). Together, these expression profiles indicated that the miPS cell lines were pluripotent since they could give rise to differentiated cells expressing markers of each of the three germ layers.

### Targeted differentiation of miPS cells towards mesenchymal stem cells

**Figure 5:**
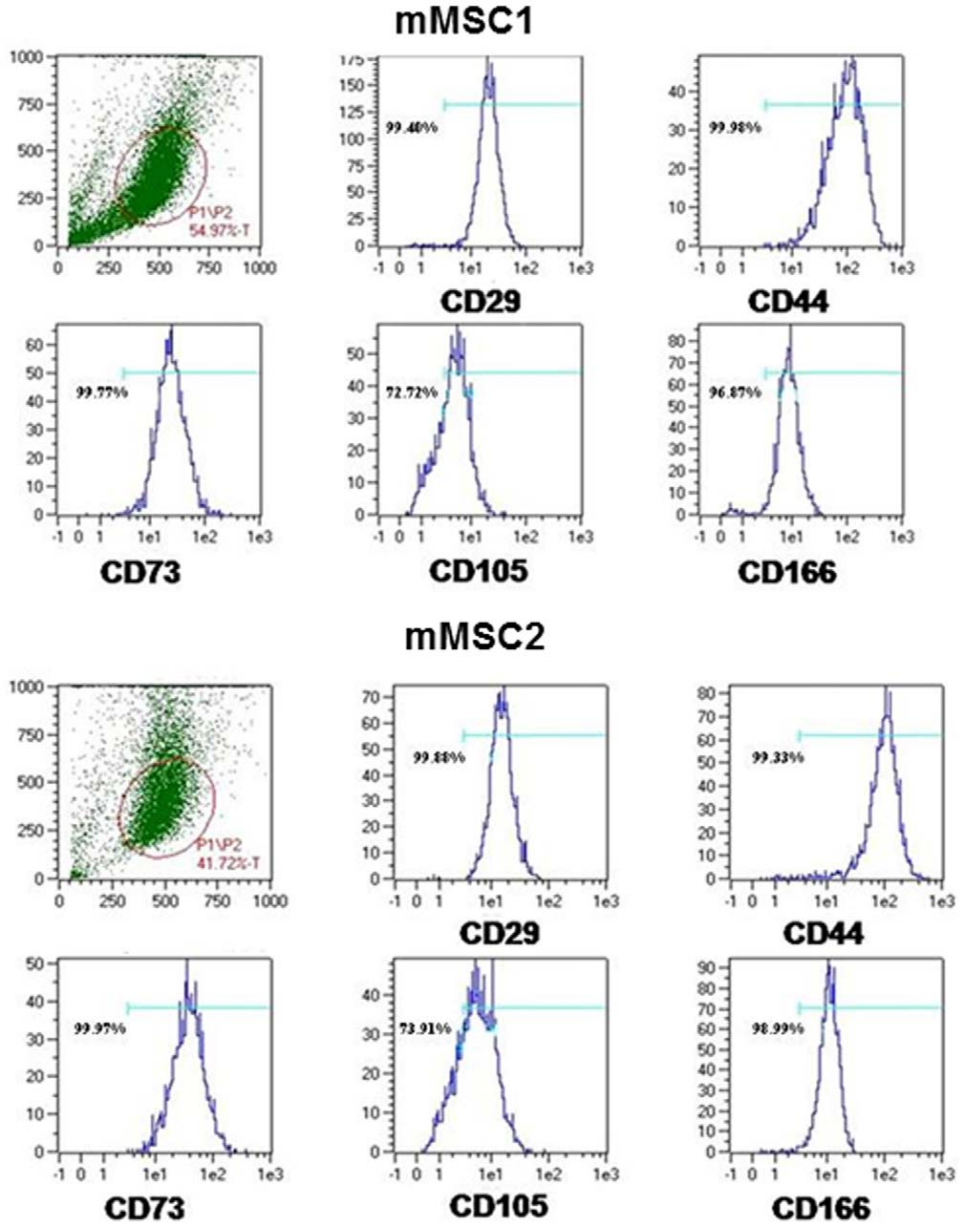
Characterization of mMSC1 and mMSC2 surface antigens. The presence of CD29, CD44, CD73, CD105 and CD166 was detected by FACS analysis performed at passage 5.

We next submitted miPS cells to *in vitro* directed differentiation by incubation in culture medium supplemented with FCS, FGF2 and Vitamin C as described in *Materials and Methods*. The initially round shaped cells (**Figure S3A**, passage 0, day 2) progressively acquired a more elongated phenotype consistent with an MSC morphology. This was evident by day 21 when the cell culture was first passaged (P1). Two days later at passage 2, a population of long and thin MSC designated as mMSC (miPS-derived MSC; Figure S3B) was apparent in both mMSC1 and mMSC2. At passage 5, the two cell lines analyzed by flow cytometry similarly expressed MSC markers CD29 (integrin β-1), CD44 (chondroitin sulfate proteoglycan 8), CD73 (ecto-5′-nucleotidase), CD105 (endoglin) and CD166 (activated leucocyte cell adhesion molecule) at their cell surface (**Figure 5**).

### Global gene expression analysis of genes differentially expressed in miPS cells, mMSC and myoblasts *vs* fibroblasts

**Figure 6:**
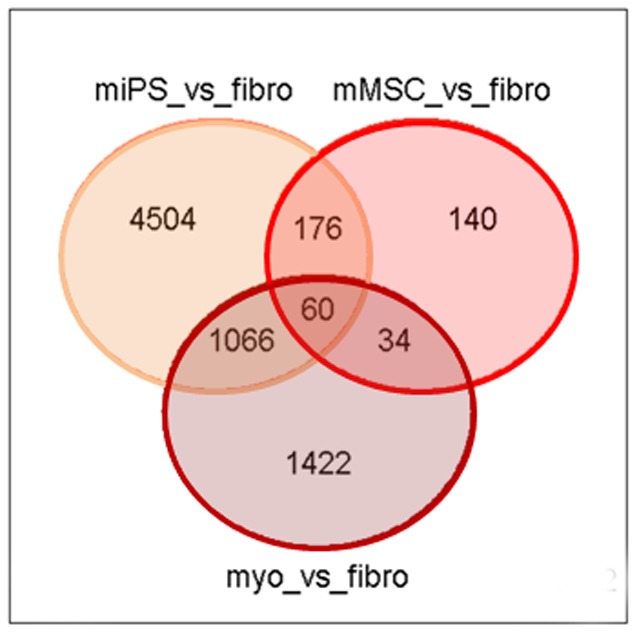
Venn diagrams. A representation of the number of genes found upregulated in cells of the myogenic lineage (myo, miPS and mMSC) in comparison with fibroblasts (fibro). The total number of unique genes analyzed after preprocessing was 17,994 with adjusted p value<0.05.

Several studies have been dedicated to a comparative characterization of iPS cells produced from cells derived from the ectodermal, mesodermal or endodermal germ layers [16] [18]. Here, we have compared gene expression profiles determined by 44K+ Agilent cDNA microarrays in miPS cells that we produced from myoblasts with the fiPS from fibroblasts that were previously produced and characterized at I-Stem (Evry, France) [19], both of mesodermal origin. In a search for a myoblast-specific signature, a functional analysis was performed along with an empirical Bayesian statistical approach to compare expression patterns in myoblasts, miPS cells and mMSC versus cells not belonging to the myogenic lineage. In the Venn Diagram exhibited in **Figure 6**, each circle represents a contrast between two expression profiles, revealing genes that have a common pattern of upregulation. Pairwise comparisons were carried out between miPS and fibroblasts (miPS_vs_fibro), mMSC and fibroblasts (mMSC_vs_fibro) and myoblasts and fibroblasts (myo_vs_fibro). Among the 17,994 genes analyzed, 2,582 (14.3%) were more expressed in myoblasts than in fibroblasts (**Figure 6**, lower circle). Among them, 1,126 (1,066+60) were expressed to a higher level in miPS cells than in fibroblasts (**Figure 6**, intersection of the lower and upper left circles). However, those represented a small fraction only of the 5,806 genes (32.3%) that were more expressed in miPS cells than in fibroblasts (upper left circle). When mMSCs were compared with fibroblasts (**Figure 6**, upper right circle), the overall number of genes differentially expressed was only 410 (2.3% of total genes). All together, there were only 60 genes (0.3% of all genes analyzed, intersection of the three circles) that were expressed to a higher level in myoblasts, miPS cells and mMSC as compared to fibroblasts.

In order to see the fold change differences in gene expression profiles between the myoblast and fibroblast lineages, contrast profiles were determined looking at the top 100 most differentially expressed genes in mMSC vs fMSC, miPS vs fiPS cells and myoblast vs fibroblast parental cells. While the value of the log(Odds) was maximal between myoblastic and fibroblastic parental cells, differences between the two lineages were less important at the MSC stage (**Figure S5**). Strikingly, they were even less pronounced when miPS and fiPS cells were compared (**Figure S5**), confirming the observed similarity between mMSC and fMSC in terms of gene expression.

### Functional analysis of genes more highly expressed in cells belonging to the myogenic lineage

We then turned to Gene Ontology descriptors to approach the function of the genes which were more highly expressed in the myogenic lineage than in fibroblasts. To this end, we searched our gene sets for shared system descriptors (see *Materials and Methods*). *ACTA* (alpha skeletal muscle actin), *MYH2* (myosin, heavy chain 2, skeletal muscle, adult), *TNNT1* (troponin T, slow skeletal muscle), *ACTN2* (alpha-actinin skeletal muscle), *TRIM63* (muscle-specific ring finger protein) and *RYR1* (Skeletal muscle calcium release channel) were present among the 1,126 genes that were overexpressed in both miPS and myoblasts versus fibroblasts, exhibiting a high score of muscle phenotype and muscle GO-terms such as muscle contraction, muscle filament sliding, structural constituents of muscle, myogenesis and myopathy (see list in **Table 1**). The 94 genes that were more expressed in both mMSC and myoblasts versus fibroblasts are found in **Table 2**. The four genes with muscle-specific GO-terms were *MYOZ2* (myozenin 2), *CA3* (carbonic anhydrase III, muscle specific), *PDLIM3* (actinin-associated LIM protein) and *ANKRD1* (ankyrin repeat domain 1, cardiac muscle), a set different from that identified above in the miPS cell comparison.

Thus, a limited number of muscle-specific genes have been identified as following a pattern of upregulation in myoblasts and miPS cells versus fibroblasts (3.6% of all overexpressed genes) while an even smaller number of genes exhibited the same differential pattern extended to the miPS versus fiPS contrast. Finally, no muscle-specific genes were found to be significantly upregulated in mMSC as compared to fMSC (data not shown).

### Differential expression analysis of muscle specific genes in the myoblast and fibroblast lineages across iPS, MSC and differentiated cells

**Figure 7:**
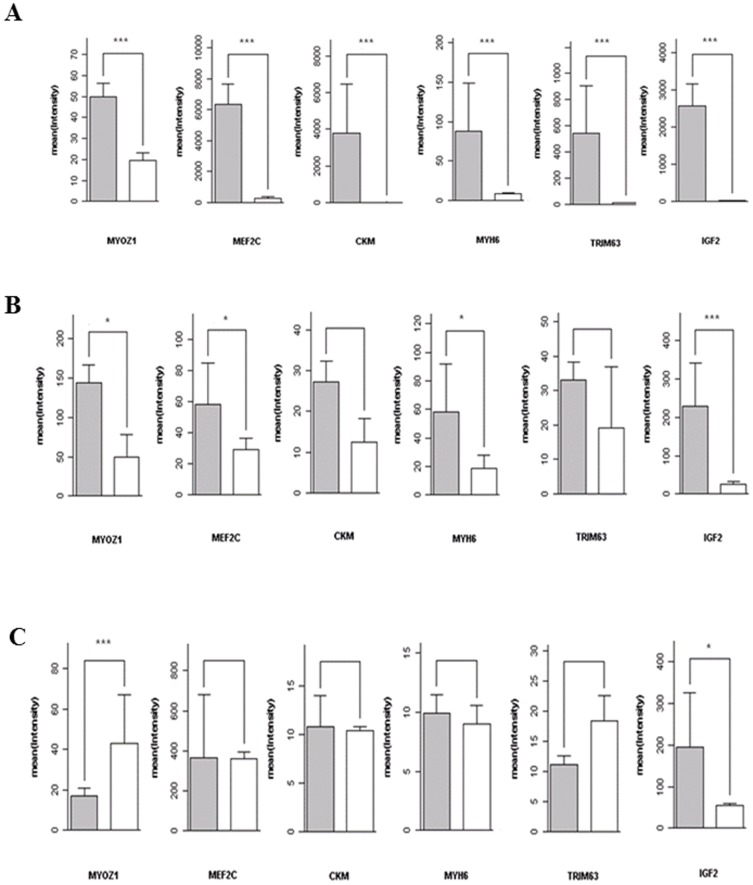
Expression of myogenic markers. The indicated markers were analyzed in in differentiated parental cells (**A**), iPS cells (**B**) and MSC (**C**) belonging to the myoblast (grey bar) and fibroblast (white bar) lineages. The experiments were carried out in duplicate. *** P<0.001, * P<0.05. Data were extracted from the 44K+ Agilent transcriptome array and scaled.

Finally, a closer look was given at the six genes, *MYOZ1, MEF2C, CKM, MYH6, TRIM63* and *IGF2* classically associated with myogenesis (**Figure 7** **, Figure S4**) [23]. As expected, all six were more highly expressed in miPS than in fiPS cells (**Figure 7** **and Table S4**) : *myosin* (*MYH6*) (p<0.05, logFC = 3.0), *creatine kinase* (*CKM*) (p = 0.1, logFC = 2.36), *MEF2C* (myocyte enhancer factor 2C) (p = 0.052, logFC = 1.9), *TRIM63* (tripartite motif containing 63, muscle specific ring finger protein 2) (p = 0.27, logFC = 1.2), *IGF2* (insulin-like growth factor 2) (p<0.005, logFC = 8.0) and *myozenin* (*MYOZ1*) (p = 0.056, logFC = 3.0) (**Figure 7** **, panel B and Table S4**). Five were highly expressed in parental myoblasts but not or hardly at all in parental fibroblasts (**Figure 7** **, panel A and Table S4**). Strikingly, these differences were largely lost between MSC derived from miPS or fiPS cells (**Figure 7** **, panel C, and Table S4**). *IGF2* remained better expressed in mMSC than in fMSC, but the other five genes were expressed more highly in fMSC (*MYOZ1* and *TRIM63*) or similarly in both MSC types (*CKM* and *MYH6*). These observations were reproducible within each category of cell lines (**Table S4**). Surprisingly, miPS and fiPS cells exhibited no differences in their expression levels of *MyoDl*, *myogenin*, *MRF4* and *MYF5*, the four core myogenic regulatory genes which together initiate the myogenesis process [15].

## Discussion

In this work, we have produced iPS cells from human MyoD-expressing CD56+ fully committed myogenic cells by retroviral transduction with OCT3/4, SOX2, MYC and KLF4 coding sequences. These miPS cells obtained expressed genes expected for iPS cells, including *OCT3/4* and *NANOG* which were activated as indicated by the decreased methylation of their promoters in comparison with their parental cell-lines. In addition, they were endowed with the capacity to differentiate into embryoid bodies which contained cells expressing surface markers characteristic of the three germ layers. That these miPS cells were pluripotent and functional was further demonstrated by their capacity to produce MSC which exhibited the CD29, CD44, CD73, CD105 and CD166 surface antigens but had lost expression of all stem cell markers.

From earlier studies, iPS cells are believed to retain the transcription memory of the germ layer origin of their parental somatic cells [16] [18]. It is not clear, however, whether iPS cells generated from parental cells sharing the same germ layer origin should display a common expression pattern. Here, we have carefully characterized gene expression in iPS cells produced from either myoblasts or fibroblasts, both derived from the mesoderm. As anticipated, miPS and fiPS gene expression patterns were found to reside within the same large cluster generated using an unsupervised HCA. Expression differences were less important between the four miPS cell lines than with the fiPS cell line which lay in a distinct subcluster. Interestingly, EB cells derived from one miPS cell-line and tested at either day 4 or day 14 localized to the same large cluster. The second large cluster generated from this HCA contained both myoblastic and fibroblastic parental cells which, however, mapped to distinct subclusters. Similarly, regardless of their miPS or fiPS cell derivation, all MSC lay within the same large cluster though in distinct subclusters. From this analysis of mesodermal derivatives, we conclude that the nature of the cells analyzed is less important than their stage of differentiation, e.g. miPS and fiPS cells on one hand, or mMSC and fMSC on the other hand, have more in common than with their parental myoblasts or fibroblasts, respectively.

The expression of several muscle-specific marker genes (actins, myosins, creatine kinase and others) was significantly stronger in miPS than in fiPS cells (**Figure 7** **, Figure S5**). In contrast, there were no significant differences between myoblast- and fibroblast-derived MSC. From the transcriptome analysis, however, four muscle-specific genes were found upregulated in muscle-derived mesenchymal stem cells and in myoblasts: A*NKRD1* (ankyrin repeat domain 1 (cardiac muscle), *TGFB2* (transforming growth factor, beta 2), *PDGFA* (Platelet-derived growth factor A chain) and *PDLIM3* (Alpha-actinin-2-associated LIM protein). Two of them, *ANKRD1* and *TGFB2*, are highly expressed in mMSCs as in fMSCs. The PDGFA and PDLIM3 are highly expressed only in muscle lineage derivatives, miPS, mMSC and myoblasts. The PDGFA protein, the main determinant of alpha-actin filament polymerization, upregulates smooth muscle alpha-actin expression [24]. This factor mitogenic for cells of mesenchymal origin is also known as a paraxial mesodermal marker and has recently been proposed as a tool to sort mesenchymal cells with a substantial muscle regeneration potential [25], [26]. PDLIM3 is a muscle specific protein involved in cytoskeletal assembly (actin filament organization) in skeletal muscles [27].

In conclusion, we have successfully generated and characterized iPS cells from human primary myoblasts and we have produced MSC from these miPS.

## Supporting Information

Figure S1 **miPS cell lines derived from myoblasts maintain normal karyotypes at passage 22.** Chromosomal contents were analyzed with high resolution G-banding technique.
(TIF)

Figure S2 **Retroviral transgene control analysis by RT-PCR for miPS cells derived from myoblasts (P: parental cells, miPS cells: clones 7 and 11, EB: miPS cells differentiated into embryoid bodies, MSC : mesenchymal stem cells derived from miPS cells, HK: housekeeping gene β-actin).** The experiments were carried out in duplicate.
(TIF)

Figure S3 **Microscopic observations of A, human mMSC at various times of differentiation (scale bar, 50 µm); B, enlarged view of MSC1 line at day 8 passage 0.**
(TIF)

Figure S4 **Expression of myogenic markers in myoblast (grey bar) and fibroblast (white bar) individual cell line lines across parental cells (A), iPS cells (B) and MSC (C).** The experiments were carried out in duplicate.
(TIF)

Figure S5 **Different log (Odds) change in expression pattern between histological (myo-fibro) contrasts across parental cells (P), iPS cells and MSC.** Distribution of log(Odds) for the first 100 most significant probes, P<0.05. Odds = prob(diff_exp)/prob(not_diff_exp). *OY* – density of genes differentially expressed when myoblast lineage was compared to fibroblast lineage, *OX* – fold change in log(Odds) of difference of gene expression between myoblast and fibroblast lineages.
(TIF)

Table S1 **List of cell lines used.**
(DOCX)

Table S2 **Primer sequences used for PCR amplification for Bisulfite Pyrosequencing Analysis.**
(DOCX)

Table S3 **Primer sequences used for RT-PCR amplification.**
(DOCX)

Table S4 **Muscle-specific genes with a positive trend of the miPS/fiPS fold change. Two comparisons are shown (i) one fiPS grown on human feeder against four miPS and (ii) one fiPS grown on human feeder + two fiPS grown on murine feeder against 4 miPS.**
(DOCX)
