# Supplementary figures and images for: Differences in Transcription Patterns between Induced Pluripotent Stem Cells Produced from the Same Germ Layer Are Erased upon Differentiation
Source: PLoS One. 2013 Jan 9;8(1):e53033. doi: 10.1371/journal.pone.0053033 (PMC3541362; doi:10.1371/journal.pone.0053033)

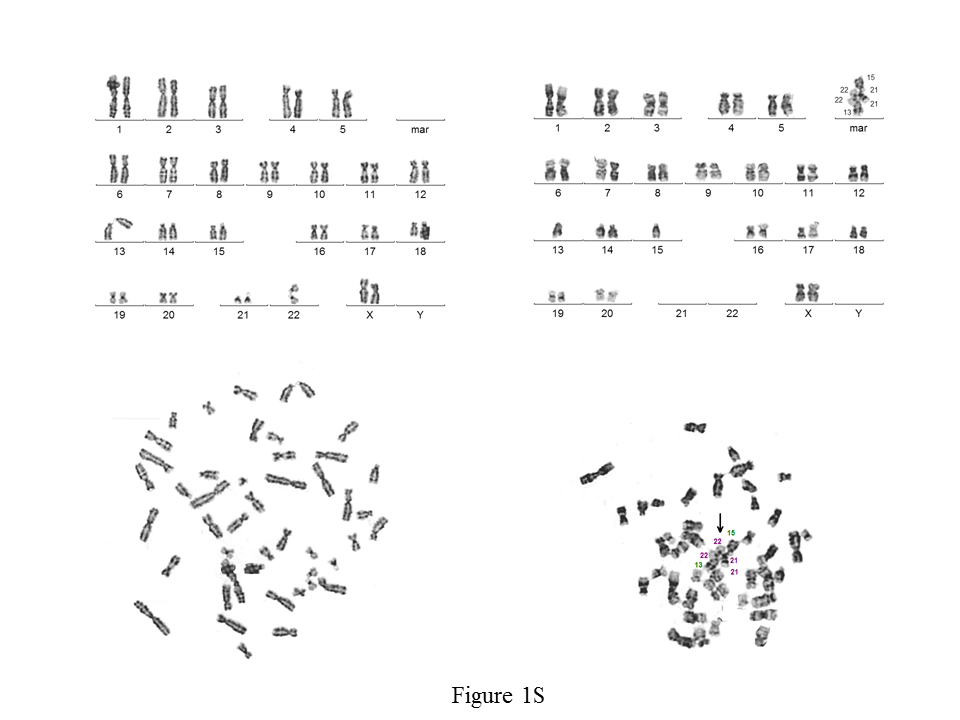

Supplement: Figure S1 — miPS cell lines derived from myoblasts maintain normal karyotypes at passage 22. Chromosomal contents were analyzed with high resolution G-banding technique. (TIF) [file pone.0053033.s001.tif]

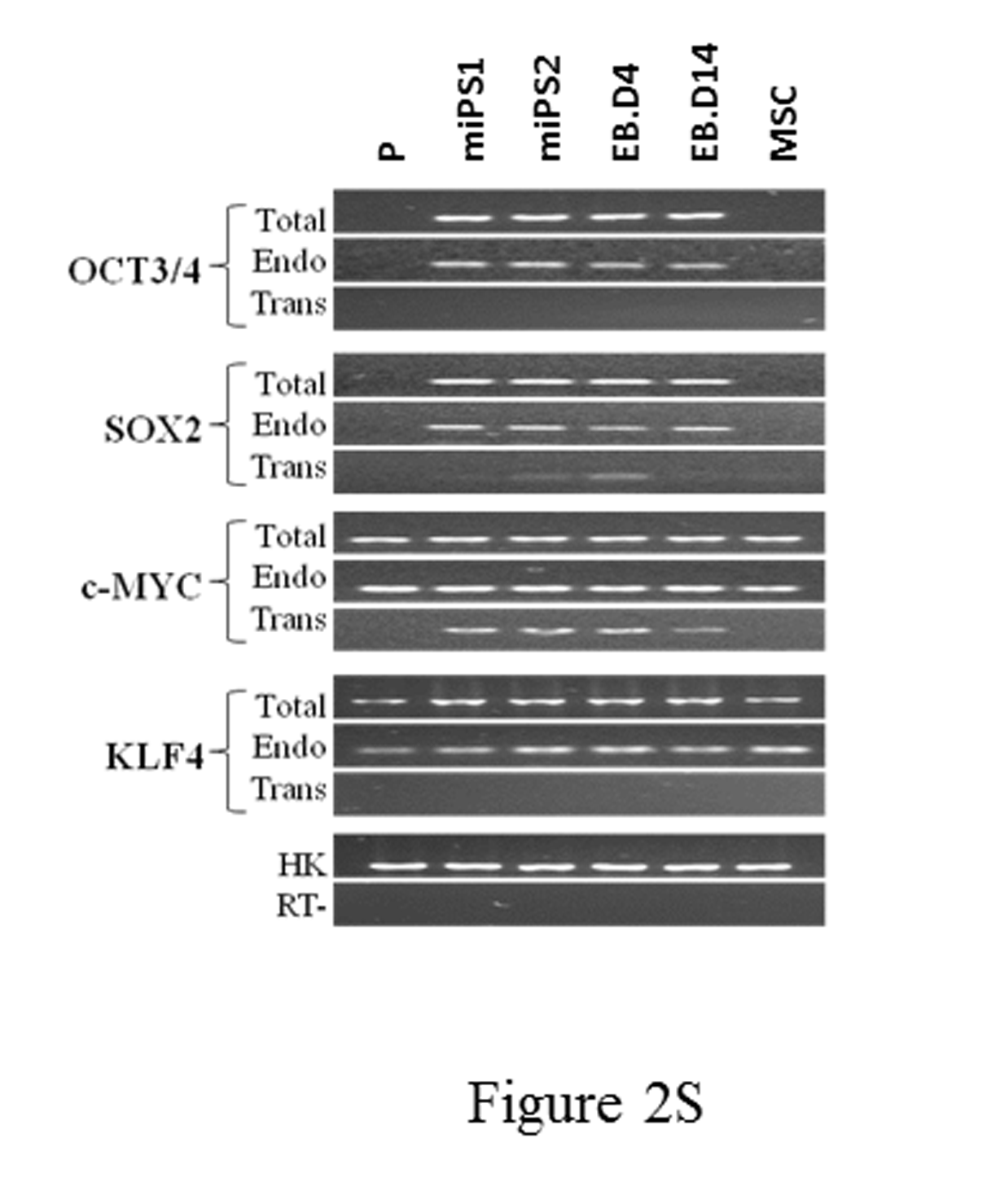

Supplement: Figure S2 — Retroviral transgene control analysis by RT-PCR for miPS cells derived from myoblasts (P: parental cells, miPS cells: clones 7 and 11, EB: miPS cells differentiated into embryoid bodies, MSC : mesenchymal stem cells derived from miPS cells, HK: housekeeping gene β-actin). The experiments were carried out in duplicate. (TIF) [file pone.0053033.s002.tif]

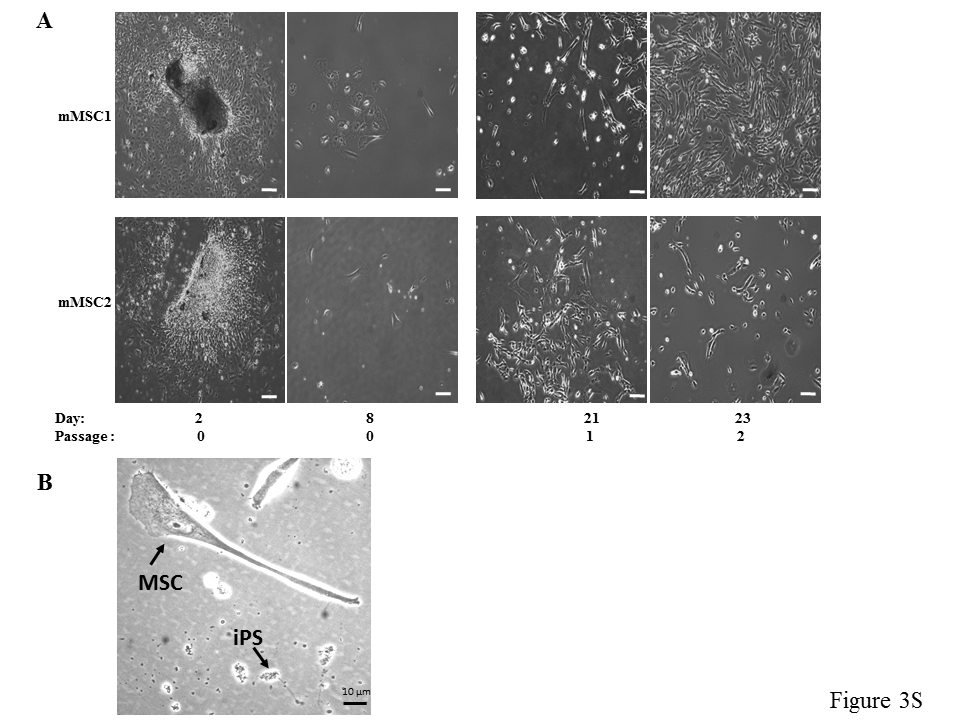

Supplement: Figure S3 — Microscopic observations of A, human mMSC at various times of differentiation (scale bar, 50 µm); B, enlarged view of MSC1 line at day 8 passage 0. (TIF) [file pone.0053033.s003.tif]

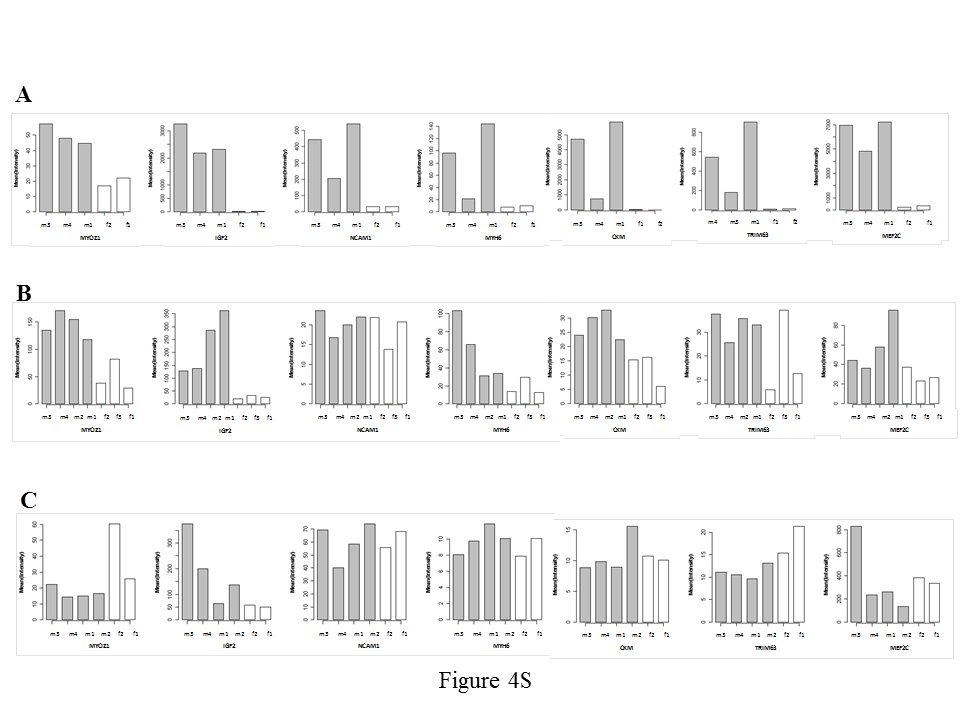

Supplement: Figure S4 — Expression of myogenic markers in myoblast (grey bar) and fibroblast (white bar) individual cell line lines across parental cells (A), iPS cells (B) and MSC (C). The experiments were carried out in duplicate. (TIF) [file pone.0053033.s004.tif]

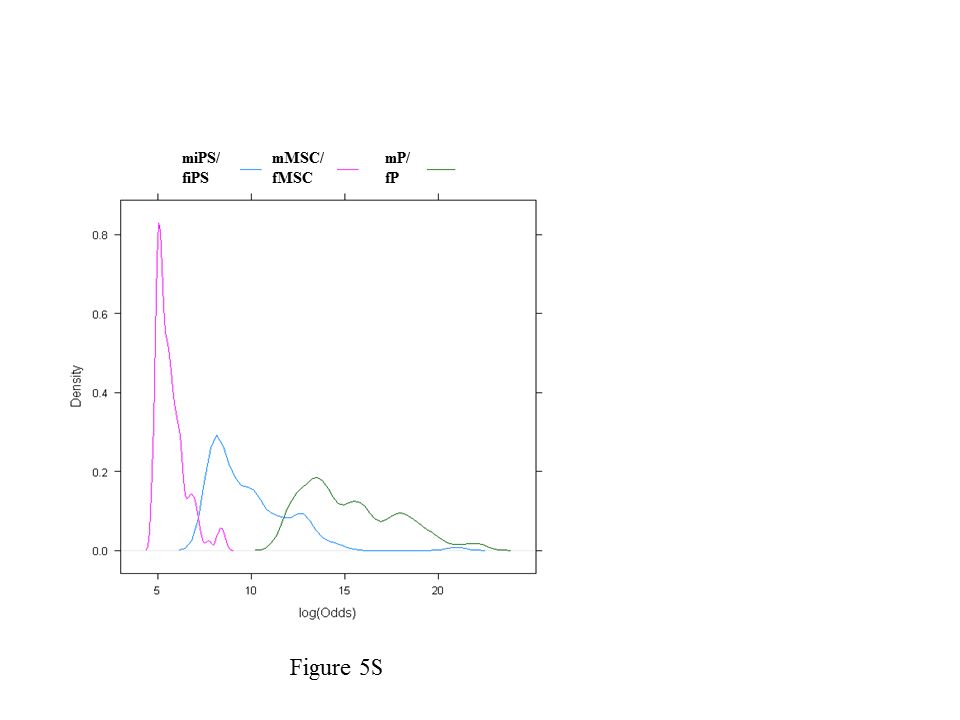

Supplement: Figure S5 — Different log (Odds) change in expression pattern between histological (myo-fibro) contrasts across parental cells (P), iPS cells and MSC. Distribution of log(Odds) for the first 100 most significant probes, P<0.05. Odds = prob(diff_exp)/prob(not_diff_exp). OY – density of genes differentially expressed when myoblast lineage was compared to fibroblast lineage, OX – fold change in log(Odds) of difference of gene expression between myoblast and fibroblast lineages. (TIF) [file pone.0053033.s005.tif]
