# Supplementary material for: Differences in Transcription Patterns between Induced Pluripotent Stem Cells Produced from the Same Germ Layer Are Erased upon Differentiation
Source: PLoS One. 2013 Jan 9;8(1):e53033. doi: 10.1371/journal.pone.0053033 (PMC3541362; doi:10.1371/journal.pone.0053033)
Supplement: Table S1 — List of cell lines used. (DOCX) [file pone.0053033.s006.docx]

Table S1. List of cell lines used

|  | Clone ID | Origin | Reference |
| --- | --- | --- | --- |
| Induced pluripotent stem cells from myoblasts | miPS1 | mP1 | This work |
|  | miPS2 | mP1 | This work |
|  | miPS3 | mP3 | This work |
|  | miPS4 | mP4 | This work |
| Induced pluripotent stem cells from fibroblasts | fiPS1 | fP1 | [19] |
|  | fiPS2 | fP2 | [19] |
|  | fiPS3 | fP1 | [19] |
| Mesenchymal stem cells from myoblasts | mMSC1 | miPS1 | This work |
|  | mMSC2 | miPS2 | This work |
|  | mMSC3 | miPS3 | This work |
|  | mMSC4 | miPS4 | This work |
| Mesenchymal stem cells from fibroblasts | fMSC1 | fiPS1 | This work |
|  | fMSC2 | fiPS2 | This work |
| Embryoid bodies from myoblasts | mEB1 | miPS1 | This work |
|  | mEB2 | miPS2 | This work |
| Myoblast parental cells | mP1 | Human myoblast culture | [14] |
|  | mP3 | Human myoblast culture | [14] |
|  | mP4 | Human myoblast culture | [14] |
| Fibroblast parental cells | fP1 | Human fibroblast culture | [19] |
|  | fP2 | Human fibroblast culture | [19] |
| Feeders | BJ1 | Human GFP fibroblast feeder cells | [19] |
|  | MEF | Mouse embryonic fibroblast feeder cells | [19] |
