# Supplementary material for: Differences in Transcription Patterns between Induced Pluripotent Stem Cells Produced from the Same Germ Layer Are Erased upon Differentiation
Source: PLoS One. 2013 Jan 9;8(1):e53033. doi: 10.1371/journal.pone.0053033 (PMC3541362; doi:10.1371/journal.pone.0053033)
Supplement: Table S2 — Primer sequences used for PCR amplification for Bisulfite Pyrosequencing Analysis. (DOCX) [file pone.0053033.s007.docx]

**Table S2. Primer sequences used for PCR amplification for Bisulfite Pyrosequencing Analysis**

| **Gene** | **Size** | **PCR primer forward** | **PCR primer reverse** | **Pyrosequencing primer(s)** | **CpGs** |
| --- | --- | --- | --- | --- | --- |
| **OCT4 /POU5F1** **(NM_002701.4) chr6:31,138,484-31,138,780** | **297** | **GAAGGATTGTTTTGGTTTAGTAGAT** | **Biotine-CACCCACTAACCTTAACCTCTAAC** | **TTGTATTGAGGTTTTGGA** | **2-4** |
|  |  |  |  | **ATTGTTATTATTATTAGGTA** | **4-5** |
| **OCT4 /POU5F1** **(NM_002701.4) chr6:31,138,341-31,138,510** | **170** | **GGGGTTAGAGGTTAAGGTTAGTG** | **Biotine-ACCTCCACCACCTAAAAAAAAC** | **GAGAGAGGGGTTGAGT** | **1-3** |
|  |  |  |  | **TTTATTTTATTAGGTTTT** | **4-7** |
| **NANOG** **(NM_024865) chr12:7,941,618-7,941,947** | **330** | **GGTTTTAAATTTTTGATTTTAGGTGATT** | **Biotine-TACTAACCCACCCTTATAAATTCTCAATTA** | **TTTTGATTTTAGGTGATT** | **1-2** |
|  |  |  |  | **TTAATTTATTGGGATTATAG** | **3-6** |
|  |  |  |  | **TTTAGAAGTATTTGTTGTTG** | **7** |
|  |  |  |  | **TTTGGTGAGATTGGTAGA** | **8** |
|  |  |  |  | **GTYGTTTAGGTGTTA** | **25-35** |
