# Supplementary material for: Differences in Transcription Patterns between Induced Pluripotent Stem Cells Produced from the Same Germ Layer Are Erased upon Differentiation
Source: PLoS One. 2013 Jan 9;8(1):e53033. doi: 10.1371/journal.pone.0053033 (PMC3541362; doi:10.1371/journal.pone.0053033)
Supplement: Table S3 — Primer sequences used for RT-PCR amplification. (DOCX) [file pone.0053033.s008.docx]

Table S3. Primer sequences used for RT-PCR amplification

| Gene | GCID | PCR primer forward | PCR primer reverse |
| --- | --- | --- | --- |
| AFP | GC04P074291 | GAATGCTGCAAACTGACCACGCTGGAAC | TGGCATTCAAGAGGGTTTTCAGTCTGGA |
| T/brachyury | GC06M166541 | GCCCTCTCCCTCCCCTCCACGCACAG | CGGCGCCGTTGCTCACAGACCACAGG |
| TnTc/TNNT2 | GC01M201328 | ATGAGCGGGAGAAGGAGCGGCAGAAC | TCAATGGCCAGCACCTTCCTCCTCTC |
| PAX6 | GC11M031768 | ACCCATTATCCAGATGTGTTTGCCCGAG | ATGGTGAAGCTGGGCATAGGCGGCAG |
| MAP2 | GC02P210252 | CAGGTGGCGGACGTGTGAAAATTGAGAGTG | CACGCTGGATCTGCCTGGGGACTGTG |
| t-OCT3/4 | total *OCT3/4* | AGCGAACCAGTATCGAGA | TTACAGAACCACACTCGGAC |
| e-OCT3/4/ POU5F1 | GC06M031136 | CCTCACTTCACTGCACTGTA | CAGGTTTTCTTTCCCTAGCT |
| tr-OCT3/4 | transgene *OCT3/4* | CCTCACTTCACTGCACTGTA | CCTTGAGGTACCAGAGATCT |
| t-SOX2 | total *SOX2* | AGCTACAGCATGATGCAGGA | GGTCATGGAGTTGTACTGC |
| e-SOX2 | GC03P181429 | CCCAGCAGACTTCACATGT | CCTCCCATTTCCCTCGTTTT |
| tr-SOX2 | transgene *SOX2* | CCCAGCAGACTTCACATGT | CCTTGAGGTACCAGAGATCT |
| t-MYC | total *MYC* | ACTCTGAGGAGGAACAAGAA | TGGAGACGTGGCACCTCTT |
| e-MYC | GC08P128748 | CATCCTGTCCGTCCAAGCA | CCGTAGCTGTTCAAGTTTGTGTTT |
| tr-MYC | transgene *MYC* | TGCCTCAAATTGGACTTTGG | CGCTCGAGGTTAACGAATT |
| t-KLF4 | total *KLF4* | TCTCAAGGCACACCTGCGAA | TAGTGCCTGGTCAGTTCATC |
| e-KLF4 | GC09M110247 | GATGAACTGACCAGGCACTA | GTGGGTCATACCACTGTCT |
| tr-KLF4 | transgene *KLF4* | GATGAACTGACCAGGCACTA | CCTTGAGGTACCAGAGATCT |
| Β-actine/ACTB | GC07M005566 | AGAAAATCTGGCACCACAC | CTCCTTAATGTCACGCACG |
