# Supplementary material for: Differences in Transcription Patterns between Induced Pluripotent Stem Cells Produced from the Same Germ Layer Are Erased upon Differentiation
Source: PLoS One. 2013 Jan 9;8(1):e53033. doi: 10.1371/journal.pone.0053033 (PMC3541362; doi:10.1371/journal.pone.0053033)
Supplement: Table S4 — Muscle-specific genes with a positive trend of the miPS/fiPS fold change. Two comparisons are shown (i) one fiPS grown on human feeder against four miPS and (ii) one fiPS grown on human feeder + two fiPS grown on murine feeder against 4 miPS. (DOCX) [file pone.0053033.s009.docx]

Table S4. Muscle-specific genes with a positive trend of the miPS/fiPS fold change. Two comparisons are shown (i) one fiPS grown on human feeder agains four miPS and (ii) one fiPS grown on human feeder + two fiPS grown on murine feeder against 4 miPS.

| gene | Compared are 1 BJ1_fiPS to 4 miPS | | | Compared are 1 BJ_fiPS and 2 MEF_fiPS to 4 miPS | |
| --- | --- | --- | --- | --- | --- |
|  | FC | Moderated t-test raw.p-value | adj.p-value (multiple testing adjusted) | FC | p-value (classical t-test) |
| MYOZ1 | 1.7 | 0.053 | 0.2 | 3 | 0.056 |
| IGF2 | 5 | 0.047 | 0.0043 | 8 | 0.0013 |
| MYH6 | 1.74 | 0.34 | 0.61 | 3 | 0.039 |
| CKM | 1.7 | 0.37 | 0.63 | 2.36 | 0.108 |
| TRIM63 | 1 | 0.66 | 0.84 | 1.19 | 0.27 |
| MEF2C | 2.3 | 0.11 | 0.34 | 1.9 | 0.052 |
